# Impulsivity and compulsivity in compulsive buying

**DOI:** 10.3389/fpsyt.2025.1665182

**Published:** 2025-09-03

**Authors:** Sapir Eliyahu, Shir Rahamim, Noy Natan, Aviv M. Weinstein

**Affiliations:** Psychology Department, Ariel University, Ariel, Israel

**Keywords:** compulsive buying behavior, impulsivity, delay discounting, response inhibition, selective attention

## Abstract

**Introduction:**

The current study examined impulsivity and compulsivity in individuals with compulsive buying (CB) and those without CB.

**Methods:**

The sample consisted of 120 participants (97 women, 23 men), undergraduate psychology students (age: M = 24.5, SD = 3.9). Participants were divided into two groups: individuals who scored high on a compulsive buying scale (CBS) and individuals who scored low on the CBS. Questionnaires measured impulsivity (Barratt Impulsiveness Scale) and compulsivity (Yale-Brown Obsessive-Compulsive Scale), along with the computerized experiential delay discounting task (EDT), response inhibition (Go/No-Go), and selective attention (Dot-Probe).

**Results:**

The questionnaires indicated higher levels of impulsivity and compulsivity in the experimental group. Secondly, compulsive buyers exhibited higher error rates in the No-Go commission condition during the second part of the Go/No-Go task, indicating an impairment in response inhibition. No differences were found in delay discounting or selective attention measured by the Dot-Probe task. Additionally, compulsive buying scores positively correlated with state anxiety scores. In a following experiment, 40 students were divided into two groups: individuals with high CBS scores and individuals with low CBS scores. They performed a simulated shopping experiment using the ASOS shopping website, with their responses recorded on Zoom. Compulsive buyers were quicker to add items to the shopping cart and spent more money than non-compulsive buyers. Compulsive buying scores were also associated with impulsivity and sensation-seeking scores. Impulsivity, but not sensation-seeking, contributed to the variance of compulsive buying scores.

**Discussion:**

This study showed impaired inhibition with a higher cognitive load on the Go/No-Go task in compulsive buyers. Secondly, there was evidence for impulsivity indicated by negative correlations between compulsive buying scores and reaction times on the in the No-Go commission condition on the Go/No-Go task and on the Dot-Probe task. Finally, in a simulation of real-life shopping, compulsive buyers were faster to choose items, and they paid more for them. This evidence suggests that the effects of compulsive buying on cognitive function are often subtle and a real-life simulation that uniquely demonstrates this impairment.

## 1 Introduction

Shopping and buying products is a daily routine that can be done either physically or online, using computers or mobile phones. The high accessibility of online shopping may encourage individuals to consume more products than they normally do, and some individuals display pathological behaviors, including the urge to buy unnecessary products, overspend, hide, or give them away as presents (1). Müller et al. (2) defined this behavior as excessive buying, characterized by frequent shopping or buying urges that overcome an individual and are perceived as irresistible drives to buy Compulsive buying is frequently comorbid with psychiatric disorders. Approximately 32% of individuals with depression exhibit compulsive buying behaviors, and 44% of compulsive buyers meet criteria for anxiety disorders (3, 4). Comorbidity is also observed with obsessive-compulsive disorder (OCD), eating disorders, and familial alcohol use disorders (5, 6). Moreover, trait anxiety has been linked to compulsive buying and OCD symptoms (7). Epidemiological meta-analyses estimate the prevalence of compulsive buying disorder (CBD) at approximately 4.9% in the general adult population, with higher rates among university students (8.3%) (8). These studies report no significant sex differences in CBD prevalence, and factors such as marital status and ethnicity do not significantly influence disorder onset. Instead, lower income and younger age—typically late adolescence to early adulthood, when individuals gain financial independence—are identified as risk factors (1, 9). While compulsive buying is most often reported in Western countries, higher prevalence rates have also been documented in East Asia (e.g., China, Taiwan, South Korea) and developing countries such as Iran (1). Many compulsive buyers report feelings of anxiety and depression that lead to psychological despair before buying. The buying relieves this distress, and it is rewarding, but it is followed by regret and guilt due to the incompatibility of this behavior with the buyer’s circumstances and its negative consequences. Currently, the DSM-5-TR (10) does not include compulsive buying as a clinical disorder. The ICD-11 (11) has classified compulsive buying/shopping disorder among other specified impulse control disorders, coded as 6C7Y. There are two terms for this phenomenon: either Buying Shopping Disorder (BSD), which implies excessive preoccupation with buying or the urge to buy (12), or Compulsive Buying Disorder (CBS), which is characterized by irresistible frequent buying in response to repeated urges to buy things that are often unnecessary. The ICD-11 defines impulse control disorders as continuous failure to resist the urge to perform a rewarding act in the short run, despite the long-term damage caused to oneself or others (11). However, Müller et al. (13, 14) and other experts argued that pathological buying is an impulse control disorder due to addictive behavior and should be included in the section of other disorders related to addictive behavior that pertains to substance use and adjunct disorders, coded as 6C5Y. These theoretical and clinical differences emphasize the lack of consensus in the area of whether compulsive BSD is an addictive behavior with irresistible urges (12) or a narrower focus on the compulsive aspect as part of an impulse control disorder. The behavioral addiction model offers a wider scope of the psychological, social, and economic aspects of the behavior (15).

Although it may result in difficulties in creating precise diagnostic criteria due to the different theoretical models regarding BSD as an impulse control disorder (ICD-11) or a behavioral addiction (12, 13), it is important to investigate the compulsive and impulsive components of BSD. A recent review on the experimental studies concerning cognitive functions in Compulsive Buying/Shopping Disorder (CBSD) argued that the evidence supports the model of addictive behaviors (16). The results showed similar findings of cue reactivity and disadvantageous decision-making, which are found in other addictive behaviors; however, there was no strong evidence for an impairment of executive function in BSD. Few studies have shown a lack of evidence or conflicting evidence for impairment of delay discounting, Stroop, trail making, Iowa gambling task (IGT), go/no-go, and stop-signal tasks in individuals with BSD.

Impaired inhibition on the Go/No-Go Task was related to more symptoms of CBSD (17, 18). Studies involving the delay discounting task (DDT) by Nicolai and Moshagen (19) or the Cambridge Gambling Task (20) also demonstrated decision-making deficits related to CBSD. A study using the dot-probe paradigm with shopping-related compared to neutral pictures did not reveal an impaired performance in CBSD (21), and there was no correlation between CBSD and performance on the dot-probe task with shopping-related pictures (22, 23). Although some tasks, such as the DDT, Go/No-Go task, and IGT, showed an association with CBSD, Thomas et al. (16) suggested that further experimental work on affective, cognitive, and neural networks in CBSD is required, particularly in areas such as attentional bias, inhibitory control, and implicit associations.

Attentional bias is a key mechanism in addictions, where cues linked to the addictive behavior capture attention and trigger craving (24). This process may be conscious or automatic (25), forming a cycle in which craving and attention reinforce each other. These findings support the relevance of studying attentional bias in Compulsive Buying/Shopping Disorder.

Compulsivity is less well-defined and investigated than impulsivity. Few studies measure compulsive behavior using cognitive tasks in psychiatric disorders. Neurocognitive measures of compulsivity assess the ability to adapt behavior flexibly after negative feedback (on probabilistic reversal learning tasks) or switch attention between stimuli (on a set-shifting task). The diminished ability to disengage from repetitive acts or obsessive thoughts could be indicated by an impaired ability to shift sets (26). Previous studies by Derbyshire et al. (20) using the Intra-Extra Dimensional Set Shift Task to measure rule learning and behavior flexibility, Black et al. (27), and Trotzke et al. (28) who used the Wisconsin Card Sorting Test to assess categorization and cognitive shifting abilities, failed to find any impairment in individuals with CBSD.

Given the gap in our knowledge of cognitive processes associated with BSD and the two theoretical models (impulse control disorder *vs*. behavioral addiction), this study aims to assess the impulsive and compulsive characteristics of BSD using questionnaires and computerized cognitive tasks assessing executive function, including inhibitory control, delayed gratification, and attentional bias. Due to the paucity of research using computerized tasks to measure compulsivity, we have decided to focus on impulsivity and attentional bias.

It was hypothesized that:

1. Individuals with BSD will show high impulsivity, indicated by subjective ratings on the Barratt Impulsiveness Scale (BIS), the experiential delay discounting task (EDT) (29), the Go/No-Go task (30), and the simulated online shopping experiment.
2. Individuals with BSD will show high compulsivity, indicated by subjective ratings on the Yale-Brown Obsessive Compulsive Scale (31).
3. Individuals with BSD will show selective attention to shopping-buying stimuli on the dot-probe task.
4. Individuals with BSD will show higher scores on Beck Depression Inventory (BDI) (32) and the Spielberger Trait and State Anxiety Inventory (STAI) (33) than individuals with non-compulsive buying disorder.

## 2 Experiment one- impulsivity, compulsivity and attentional bias

### 2.1 Methods

#### 2.1.1 Participants

A hundred and twenty participants were recruited for this study, 97 women and 23 men. They were divided into a group of 60 participants who were classified as Compulsive Shopping Behavior (CSB) (score above 42 which is 2 SD above mean) on the Compulsive Buying Scale (34) with a mean age of 24 years and 8 months (SD = 4.3) and a group of 60 participants who were classified without CSB with scores lower than 42 on the CBS with a mean age of 24 years and 2 months (SD = 3.6). Participants were students recruited from social networks. They filled out questionnaires online. Exclusion criteria were individuals who do not use SNS, are diagnosed with ADHD, or have mental or neurological illnesses, or underage. Four participants were excluded from the original sample of 124. Seventy-five % of the sample reported below average income, 15% average income, and 10% above average income. A comparison of sociodemographic variables and age has shown no group differences. A sample size of 120 participants that are equally divided by a general recommendation that the number of participants should be at least 5 times the variables (35). In this study, there were 6 variables: compulsive buying, impulsivity, compulsivity, selective attention, anxiety, and depression. The sample was therefore large enough to provide statistical power at the 5% level.

### 2.2 Questionnaires

#### 2.2.1 Demographic questionnaire

The demographic questionnaire included questions about sex, age, education, marital status, urban living, and employment. **Table 1** shows demographic details of all participants.

**Table 1:** Demographic questionnaire ratings of the study groups (n=120).

Variables			Group	
			Compulsive buyers	Non- compulsive buyers
Age			24.81 (4.32)	24.2 (3.6)
Gender	Female	n	52	45
		%	86.67%	75%
	Male	n	8	15
		%	13%	25%
Religious	Jews	n	58	59
		%	96.7%	98.3%
	Christian	n	2	0
		%	3.3%	0
	Islam	n	0	1
		%	0	1.7%
Family status	Single	n	46	45
		%	76.7%	75%
	Married	n	14	15
		%	23.3%	25%
Academic education	No	n	49	56
		%	81.7%	93.3%
	Yes	n	11	3
		%	18.3%	5%
Birth Country	Israel	n	56	48
		%	93.3%	80%
	Other	n	4	12
		%	6.7%	20%
Dominant Hand	Right	n	48	47
		%	80%	78.3%
	Left	n	12	13
		%	20%	21.7%
Income	Above Average	n	4	11
		%	6.7%	18.3%
	Average	n	9	10
		%	15%	16.7
	Below Average	n	47	39
		%	78.3%	65%

### 2.3 Compulsive Buying Scale

The Compulsive Buying Scale (34) measures 3 aspects of compulsive buying: a) tendency to spend money, b) urge to buy 3) guilt after shopping. The scale has 13 items and scores range from 1 “not at all” to 4 “agree very much” on a Likert scale. The questionnaire had been validated with a mean Cronbach internal reliability of α = 0.78 to 0.92 (34). In our study, the questionnaire had a Cronbach’s internal reliability of α=0.85. The cut-off point of 42 was determined by the model of Faber & O’Guinn (36), who calculated compulsive buying as 2 standard deviation points above average. This criterion was validated by the studies of Reisch et al. (37) and Kaur et al. (38).

### 2.4 Barratt Impulsiveness Scale

The Barratt Impulsiveness Scale BIS-11 (39) includes 30 items on a Likert scale that range from 1 (rarely/never) to 4 (almost always/always). The BIS-11 is a 30-item self-report measure, with scores ranging from 30 to 120, where higher scores indicate higher levels of impulsivity. The questionnaire had been validated among varied populations, including students, psychiatric patients, and prisoners with a mean Cronbach’s internal reliability of α=0.79-0.83 (39). In our study, the questionnaire had a Cronbach’s internal reliability of α=0.78.

### 2.5 Yale Brown Obsessive Compulsive Scale

The Yale Brown Obsessive Compulsive Scale (31) includes ten items on a Likert scale ranging from 0 (“full control”) to 4 (“no control”). Scores range between 0 and 40, including 5 items of obsessive thoughts and 5 items of compulsive behaviors. The questionnaire had been validated with a mean Cronbach internal reliability of α=0.92 (31). In our study, the questionnaire had a Cronbach’s internal reliability of α=0.78.

### 2.6 Spielberger trait and state anxiety inventory

The STAI (33) has forty items, including twenty on trait anxiety and twenty on state anxiety. Scores on a Likert scale range from 1 (“not at all”) to 4 (“agree very much”). The questionnaire had been validated with a mean Cronbach’s internal reliability of α = 0.83 for Spielberger State and α = 0.88 for Spielberger Trait (33). In our study, we used the Trait anxiety inventory, which had a Cronbach’s internal reliability of α=0.91.

### 2.7 Beck Depression Inventory

Beck Depression Inventory (BDI) (32) is a self-report measure of attitudes and symptoms of depression (32). The inventory has twenty-one items. Scores on a Likert scale are rated from 0 to 4. The BDI has high internal consistency, with Cronbach’s internal reliability of α = 0.86 and 0.81 for psychiatric and non-psychiatric populations (40). In this study, the BDI had a Cronbach’s internal reliability of α=0.88.

### 2.8 Computerized tasks

Experiential Delay Discounting Task (EDT) by Rachlin et al. (29).

The computer screen presented either 1.2 Israeli shekels that were delayed and uncertain or a lower amount than 1.2 Israeli shekels that were immediate and changeable. Participants were instructed that if they decided to cash the money, they had to press the square on which the money was shown. There were 4 blocks with 15 trials involving different delay times for every trial (1, 5, 10, 20 seconds).

Analysis of the results of the Experiential Delay Discounting Task (EDT).

The score of delay discounting was calculated by adding all choices and times of delay (41, 42). K is a free parameter that indicates the steepness of the discount curve. High k-values indicate rapid discounting (e.g., 43–45).

### 2.9 GO/NO-GO task

The GO/NO-GO task, first developed by Luria in the 1940s, is described by Gomez et al. (30). The task requires participants to respond by pressing a button when they see a “GO” sign and not to respond when they see the “NO-GO” sign. The task measures the ability to ignore or inhibit automatic and habitual responses in favor of more deliberate actions. In this experiment, the “GO” sign was the logo of “Facebook,” and the “NO-GO” sign was arrows from a traffic sign. The experiment included 100 trials that were presented for 1.5 seconds. The probability of sign presentation (“GO” or “NO-GO”) was equal, and they were presented in a random order. After 50 trials, the order was reversed, with the “GO” sign being the arrows from a traffic sign and the “NO-GO” sign being the logo of “Facebook.” The analysis included reaction times (RTs) and commission errors, which were falsely not pressing the button in “GO” trials, and omission errors, which were falsely pressing the button in “NO-GO” trials.

### 2.10 Analysis of the results on the GO/NO-GO task

The analysis included a two-way mixed repeated measures ANCOVA of the two groups of participants (Excessive and non-excessive users of SNS) of RTs, commission, and omission errors.

### 2.11 Dot Probe Task

The computerized Dot-Probe task (46) was used to test selective attention to compulsive shopping stimuli. Data collection was performed using Python-based software PsychoPy (v2023.2.2). In each condition, a focus point is displayed on the screen, followed by two different images appearing simultaneously on both sides of the screen. The subject must press the keyboard as quickly as possible according to the side on which a dot appeared after the images were presented. The purpose of the task is to test the reaction time between the condition in which the dot appeared after shopping-related images and the reaction time in the condition in which the dot appeared after a neutral stimulus. Differences in reaction times are an indication of selective attention to shopping stimuli in compulsive shoppers. In cases of selective attention, reaction time is expected to be faster in the condition in which the dot appeared after a shopping-related stimulus compared to the neutral condition.

### 2.12 Procedure

The participants were recruited by responding to an advertisement on the university website and WhatsApp groups of students. The questionnaires were filled out on Google Forms, and afterwards, the participants were assessed on the computerized tasks.

### 2.13 Ethical approval

The study was approved by the Institutional Review Board (IRB, Helsinki committee of the University). Participants signed an informed consent form.

### 2.14 Statistical and data analysis

Results were analyzed using Excel (Version 16.90; Microsoft 2024) and JASP (Version 0.18.3; 47). Pearson’s chi-squared test was used to compare demographic factors such as sex, age, education, marital status, and employment. Group comparisons were done using t-tests and one-way ANOVA for the CBS, BIS-11, YBOCS, STAI, and BDI questionnaires, and the EDT, Go/No-Go, and dot probe task. Pearson’s correlation tests were used to associate questionnaire ratings and performance on the cognitive tasks. Since there were no group differences in demographic variables, analysis of covariance was not required.

### 2.15 Power calculations

A power calculation of the mediation model was conducted using G*Power 3.1.9.7 (48, 49) based on ANOVA and Two-way mixed design repeated measures ANOVA in accordance with the relevant literature (50). For the ANOVA, an expected effect size of 0.3 with a standard statistical power of 0.8 was selected, yielding a minimal sample size of 67 participants. For the Two-way mixed design repeated measures ANOVA, for the interaction effects, an expected effect size of 0.25 with a standard statistical power of 0.8 was selected, including two groups of participants with a minimal sample size of 34 each. For within-group comparisons, an expected effect size of 0.25 with a standard statistical power of 0.8 was selected, including 2 groups of participants with a minimal sample size of 34 each.

## 3 Results

### 3.1 Questionnaire ratings

**Table 1** shows demographic data for all participants.

Compulsive buyers scored higher than those with non-compulsive buyers on the Compulsive Buying Scale (CBS), t (118) = -14.5, p<.001, *Cohen’s d* = -2.64. Compulsive buyers scored higher than non-compulsive buyers on the BIS-11 impulsivity questionnaire, t (118) = -3.95, p <.001, *Cohen’s d* = -0.72. Compulsive buyers scored higher than non-compulsive buyers on the YBOCS questionnaire using the Mann-Whitney test due to a lack of normal distribution of the data, U = 1359, p<.01, effect size =0.245.

**Table 2** shows comparisons between groups on questionnaire ratings.

**Table 2:** Differences between compulsive buyers and non-compulsive buyers in impulsiveness, obsessive compulsive behavior, compulsive buying, state-trait anxiety and depression (n=120).

Variables	CB	n	M	SD	Comparison, Cohen’s d/effect size
BIS-11	Yes	60	67.98	10.29	
	No	60	60.73	9.79	t (118)=-3.95, p<.001, -0.72
YBOCS*	Yes	60	11.78	5.62	
	No	60	9.52	5.05	U=1359, p<.01, Effect size=-0.245
CBS	Yes	60	*48.96*	*7.33*	
	No	60	*32.66*	*4.69*	*t (118)=-14,500, p<.001, -2.64*
SSAI	Yes	60	67.98	10.29	t (118)=-3.95, p<.001, -0.72
	No	60	50.36	5.9	t (118)=-1.58, p<.05, 0.32
STAI*	Yes	60	47.83	5.8	
	No	60	47.75	5.58	U=-1781, p=0.46 Effect size=-0.01
BDI*	Yes	60	8.81	6.84	
	No	60	8.23	7.36	U=-1670, p=.25 Effect size=-0.072

BIS-11, Barratt Impulsiveness Scale.

YBOCS, Yale Brown Obsessive Compulsive Scale.

CBS, Compulsive Buying Sale.

SSAI, Spielberger State Anxiety Inventory.

STAI, Spielberger State-Trait Anxiety Inventory.

BDI, Beck Depression Inventory.

*A Mann-Whitney test was performed due to a violation of the normality of the results.

### 3.2 Cognitive tasks

#### 3.2.1 EDT

**Figure 1:**
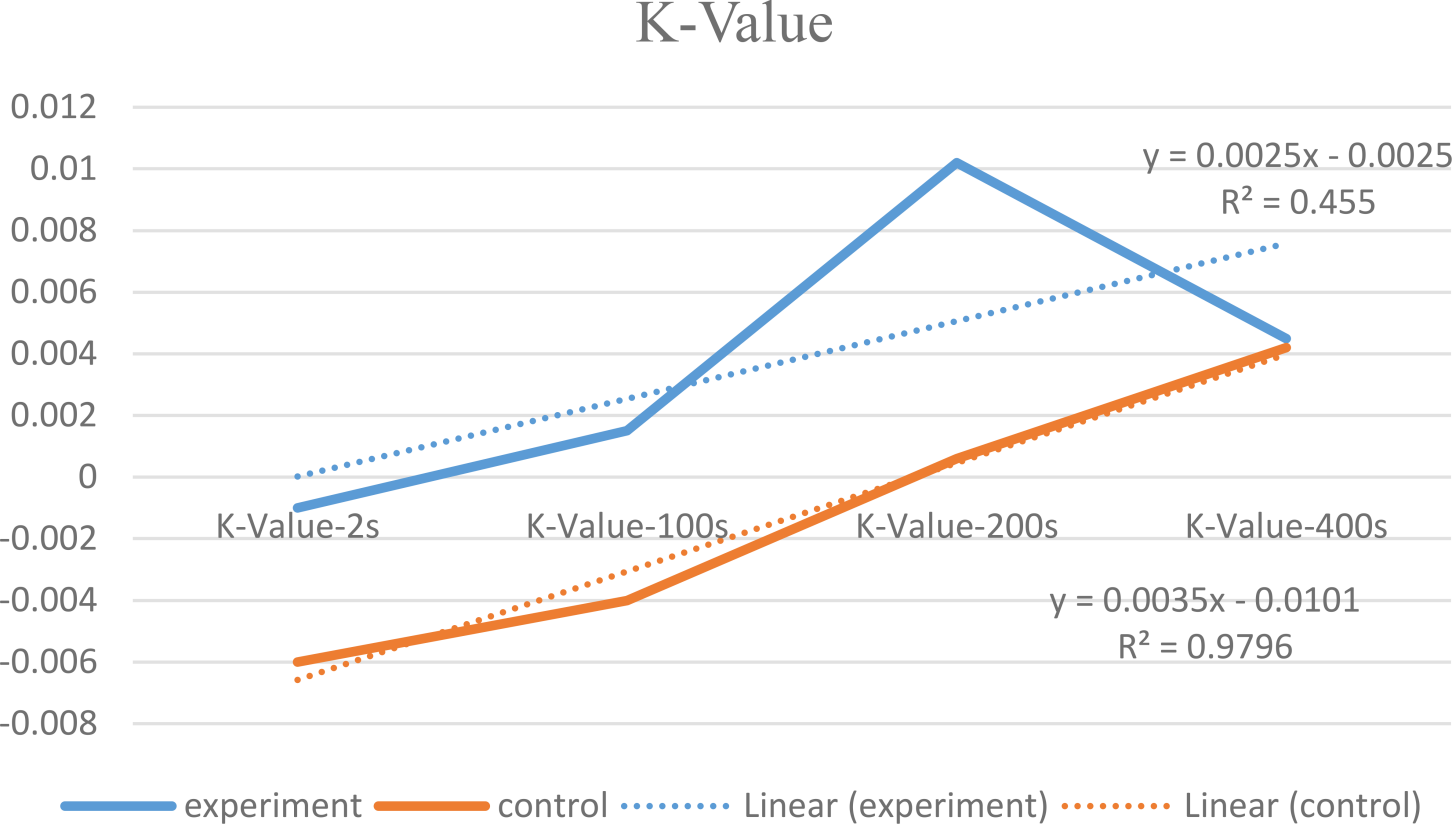
Mean group performance on the delay discounting task in compulsive and non-compulsive buyers.

For each participant, a scatter plot and a trend line that represents the relationship between variables were calculated. The X line reflects the K value, which is the slope of the delay discounting curve for each participant. Due to the lack of normal distribution of the data, a Mann-Whitney test was used to compare the experimental and control groups. The test showed no significant group difference between compulsive buyers and non-compulsive buyers on the K value, U=1847, p=0.81, Effect size= 0.03. The compulsive buying group had a y curve of y = 0.0025x − 0.0025, R^2^ = 0.45, which indicated a relatively weak correlation between K measures. The non-compulsive buying group had a y curve of y = 0.0035- 0.0101 R^2^ = 0.98, which indicated a high correlation between K measures. These findings imply that the compulsive-buyer group showed a flat delay discounting curve contrary to our prediction. **Figure 1** shows mean group performance on the delay discounting task in compulsive and non-compulsive buyers.

### 3.3 Go/No-Go

#### 3.3.1 Analysis of reaction times

A two-way mixed design repeated measures ANOVA was conducted to test the effect of group (compulsive *vs*. non-compulsive buyers) and condition (Go *vs*. No-Go) on RTs. Results reveal no effect of group (F (1, 108) = 1.41, p=.24, η^2^ = .009), a significant condition effect (F (1, 108) = 38.31, p<.001, η^2^ = .0077) and no interaction between group and condition (F (1, 108) = .73, p = .4, η^2^ = .001). The results indicate no evidence for impaired response inhibition in compulsive buyers.

### 3.4 Analysis of errors

A mixed-measures ANOVA of the error rates was conducted to test the effect of group (compulsive *vs*. non-compulsive buyers) and condition (Go *vs*. No-Go) on error rates. Results reveal an overall effect of group (F (1, 118) = 4.23, p = .042, η^2^= .021) and no effect of condition (F (1, 118) = 0.42, p = .520, η^2^= .001). There was an interaction between group and condition (F (1, 118) = 1.26, p = .263, η² = .004**).** *Post-hoc* analysis revealed that compulsive buyers did not make more errors in the Go condition compared to non-compulsive buyers (t(118) = -0.86, p = 1.000, d = -0.16), and in the No Go condition (t (118) = -2.30, p = .134, d = -0.42). The lack of significant group differences in errors in the Go and No Go conditions (omission or commission errors) indicates no evidence for impaired inhibition in compulsive buyers.

### 3.5 Further analysis

A two-way mixed design repeated measures ANOVA was conducted on the second part of the Go/No Go task to test the effect of group (compulsive *vs*. non-compulsive buyers) and condition (Go *vs*. No-Go) on RTs. Results reveal no effect of group (F (1, 78) = 2.4, p=.13, η^2^ = .018), a significant condition effect (F (1, 78) = 7.71, p<.01, η^2^ = .035) and a significant interaction between group and condition (F (1, 78) = 3.97, p<= .05, ηp^2^ = .018).

*Post-hoc* analysis revealed that compulsive buyers responded longer in the No Go condition compared to non-compulsive buyers (t (78) = 3.11, p = .016, Cohen’s d = 0.67), but not in the Go condition (t (78) = 0.018, p = 1.000, Cohen’s d = 0.004). These results indicate impaired response inhibition in compulsive buyers in the second part of the experiment.

A repeated Measures ANOVA of standard deviations between groups revealed a group effect (F (1, 43) = 7.22, p<.01, η^2^ = 0.062, a condition effect F (1.43) = 20.02 p<0.001, η² = 0.15 and a significant group and condition interaction F (1.43) = 12.26 p<0.001, η² = 0.092. These results indicate impaired performance on the response inhibition task in compulsive buyers in the second part of the experiment.

### 3.6 Dot probe task

#### 3.6.1 Initial analysis of congruence

Due to the lack of normal distribution of the data, a Wilcoxon test was conducted to compare the congruence and incongruence conditions. The analysis showed a significant difference between the congruent and incongruent conditions, U= 2189, z= 3.8, p<0.01, effect size = -0.4. This finding indicates that the participants responded faster to the congruent condition than the incongruent condition, as expected.

A mixed-measures ANOVA of the reaction times was conducted to test the effect of group (compulsive *vs*. non-compulsive buyers) and condition (congruent *vs*. incongruent) on reaction times. Results reveal an overall effect of group (F (1,118) = 4.02, p<.05, η^2^ = 0.032) and a significant effect of condition (F (1, 118) = 12.83, p<0.01, η^2^ = 0.003) and no significant interaction between group and condition (F (1, 118) = 0.07, p = 0.79, η^2^ = .00001). The results indicate no evidence for selective attention to buying pictures in compulsive buyers.

### 3.7 Analysis of errors

The experiment included online buying and offline buying, which will be analyzed separately.

A mixed-measures ANOVA was conducted to test the effect of group (compulsive *vs*. non-compulsive buyers) and condition (congruent *vs*. incongruent) on error rates in online buying. Results revealed an overall effect of group (F (1,118) = 4.02, p<.05, η^2^ = 0.032) and a significant effect of condition (F (1, 118) = 4.24, p<0.05, η^2^ = 0.001) and no significant interaction between group and condition (F (1, 118) = 0.11, p = 0.74, η^2^ = 0.001). The results indicate that compulsive buyers showed no specific attentional bias to pictures of buying items online.

A mixed-measures ANOVA was conducted to test the effect of group (compulsive *vs*. non-compulsive buyers) and condition (congruent *vs*. incongruent) on error rates in offline frontal buying. Results revealed a non-significant group effect (F (1,118) = 2.70, p=.10, η^2^ = 0.021) a significant effect of condition (F (1, 118) = 13.66, p<0.001, η^2^ = 0.006) and a non-significant interaction between group and condition (F (1, 118) = 1.78, p = 0.19, η^2^ = 0.001). The results indicate that compulsive buyers showed no specific attentional bias to pictures of buying items off-line. The results altogether indicate no evidence for selective attention to buying pictures in compulsive buyers online and offline.

### 3.8 Correlations between questionnaire ratings and performance on cognitive tasks

There were positive correlations between the CBS, BIS-11, and YBOCS questionnaires but not between questionnaires and the EDT, Go/No-Go, and dot probe task. A positive correlation was found between CBS and YBOCS ratings *r* (118) = 0.316, p <.001, which suggests that compulsive buying was associated with compulsive behavior. In addition, a negative correlation was found between CBS ratings and the average reaction time for incorrect responses (commissions errors) on the No-Go, *r* (118) = − 0.198, p< 0.05, indicating an impairment in response inhibition. CSB ratings were negatively correlated with reaction times in the matching condition on the Dot-Probe task, *r* (118) = − 0.189, p<0.05 indicating facilitated selective attention. These findings indicate that the greater compulsive buying scores, the greater the impulsivity and faster response to relevant shopping stimuli.

**Table 3** shows correlations between questionnaires and cognitive tasks.

**Table 3:** Pearson r correlations between questionnaire ratings and the computerized tasks.

Variable		CBS	Baratt	YBOCS	RT_Go_C correct	RT_NO-_Go_Wrong	Commission_ Errors_No-Go	Omission_Errors_Go	Dot-probe congruence	Dot-probe incongruence
CBS	Pearson's r	–								
	p-value	–								
BIS-11	Pearson's r	0.440***	–							
	p-value	<.001	–							
YBOCS	Pearson's r	0.316***	0.413 ***	–						
	p-value	<.001	<.001	–						
RT_Go_C correct	Pearson's r	-0.161	-0.121	-0.051	–					
	p-value	0.078	0.19	0.582	–					
RT_NO-_Go_Wrong	Pearson's r	-0.198*	-0.076	-0.04	0.614***	–				
	p-value	0.038	0.433	0.679	<.001	–				
Commission_Errors_No-Go	Pearson's r	0.007	-0.046	-0.022	-0.069	-0.074	–			
	p-value	0.938	0.615	0.811	0.455	0.441	–			
Omission_Errors_Go	Pearson's r	0.032	0.04	0.024	0.14	-0.105	0.005	–		
	p-value	0.726	0.667	0.796	0.128	0.275	0.955	–		
Dot-probe congruence	Pearson's r	-0.189*	-0.11	-0.152	0.567***	0.344***	-0.207*	0.048	–	
	p-value	0.039	0.233	0.097	<.001	< .001	0.023	0.602	–	
Dot-probe incongruence	Pearson's r	-0.151	-0.093	-0.136	0.542***	0.339***	-0.214*	0.024	0.952 ***	–
	p-value	0.1	0.314	0.138	<.001	<.001	0.019	0.796	<.001	–

*p < .05, ***p < .001.

CBS, Compulsive Buying Sale.

BIS-11, Baratt Impulsiveness Scale

YBOCS, Yale Brown Obsessive Compulsive Scale.

RT, Reaction Times.

Go, Go condition on the Go/No Go task.

No Go, No Go condition on the Go/No Go task.

### 3.9 Anxiety and depression questionnaires

The compulsive buying group scored higher on state anxiety (STAI) (M = 50.36, SD = 5.9) compared with the control group (M = 48.5, SD = 5.7), t (118) = - 1.575, p <0.05, Cohen’s d = - 0.32. A Mann-Whitney test was performed on Spielberger’s Trait Anxiety Inventory (STAI) due to the violation of the assumption of normality of the data. No difference was found between the compulsive buying group (M = 47.83, SD = 5.8) and the control group (M = 47.75, SD = 5.5), U = 1781.00, p = .461, Effect size = - 0.01. Finally, a Mann-Whitney test was also performed on depression scores (BDI) due to the violation of the assumption of normality of the data. The findings showed no difference between the compulsive buying group (M = 8.81, SD = 6.8) and the control group (M = 8.23, SD = 7.3), U = 1670.50, p = .25 effect size=-0.07. The findings indicate that compulsive buyers display higher levels of state anxiety but no trait anxiety or depression, compared to the control group.

To examine the relationship between Spielberger Trait Anxiety (STAI), State Anxiety (SSAI), and depression (BDI) and compulsive buying behavior (CSB), Pearson correlations were calculated between all variables. A positive correlation was found between SSAI rating and CSB rating (*r* (118) = 0.185, p<0.05), indicating that the higher the level of state anxiety, the higher the tendency of compulsive buying. In contrast, the correlation between depression (BDI) and compulsive buying behavior (CSB) was not significant (*r* (118) = 0.17, p = .07). In addition, a positive correlation was found between state anxiety rating (SSAI) and the STAI trait anxiety rating, *r* (118) = 0.25, p<.01, as well as a positive correlation between state anxiety (SSAI) rating and depression rating (BDI), *r* (118) = 0.57, p <.001 (see **Table 4**). These findings indicate a positive relationship between state anxiety and compulsive buying, implying that the higher the state anxiety, the higher the tendency to shop compulsively. In contrast, no relationship was found between depression and compulsive buying. In addition, there was a positive correlation between state and trait anxiety, as well as between state anxiety and depression, supporting the evidence for a strong relationship between anxiety and depression.

**Table 4:** Correlations between compulsive buying, anxiety, depression and obsessive-compulsive scale.

Variable		CBS	YBOCS	SSAI	STAI	BDI
1. CBS	Pearson's r	–				
	p-value	–				
2. YBOCS	Pearson's r	0.316***	–			
	p-value	<.001	–			
3. SSAI	Pearson's r	0.185*	0.435***	–		
	p-value	0.044	< .001	–		
4. STAI	Pearson's r	-3.741×10-4	0.037	0.244**	–	
	p-value	0.997	0.692	0.007	–	
5. BDI	Pearson's r	0.167	0.474***	0.567***	-0.046	–
	p-value	0.069	< .001	< .001	0.62	–

*p < .05, **p < .01, ***p < .001.

CBS, Compulsive Buying Scale; YBOCS, Yale–Brown Obsessive-Compulsive–Compulsive Scale; SSAI,_Spielberger State Anxiety Inventory; STAI,_Spielberger Trait Anxiety Inventory; BDI, Beck Depression Inventory.

### 3.10 Experiment 2- a simulation experiment of natural buying

The following experiment intends to simulate a real-life shopping experiment in order to examine impulsive buying online. The previous study has confirmed the association between impulsivity and compulsive buying. Sensation seeking has also been implicated in the process of compulsive buying. Sensation seeking is a personality dimension characterized by the need to seek excitement and new, various, complex and intense stimuli from the environment, and it includes a certain level of risk in physical, social and financial areas, mainly due to the experience itself (51). There is evidence for an association between compulsive buying and sensation-seeking (52, 53). In view of previous evidence for the role of impulsivity and sensation seeking in compulsive buying, the current study seeks to examine the relationship between impulsivity and sensation-seeking and compulsive online buying and to estimate the contribution of these variables to the variance of compulsive buying.

### 3.11 Hypotheses

1. Impulsivity will positively correlate with compulsive buying. Participants who buy compulsively will show a high level of impulsivity compared to non-compulsive buyers.
2. Compulsive buying will positively correlate with delayed gratification. Individuals with compulsive buying will show impaired delayed gratification.
3. A multivariate linear regression will show that the independent variables of impulsivity and sensation seeking will contribute to the variance of compulsive buying.

## 4 Methods

### 4.1 Participants

Forty participants were recruited for this study. The sample included twenty women and twenty men. They were divided into a group of 20 participants who were classified with Compulsive Shopping Behavior (CSB) (score above 42 which is 2 SD above mean) on the Compulsive Buying Scale (34) with a mean age of 24 years and 1 months (SD = 1.9) and a group of 20 participants who were classified without CSB with scores lower than 42 on the CBS with a mean age of 24 years (SD = 2.5). Participants were students, and they were recruited from social networks. They filled out questionnaires online. Exclusion criteria were individuals who do shop, are diagnosed with ADHD, or have mental or neurological illnesses, or are underage.

### 4.2 Questionnaires

#### 4.2.1 Demographic questionnaire

The demographic questionnaire includes questions about sex, age, education, marital status, urban living, and employment. **Table 5** shows demographic details of all participants. A comparison of sociodemographic variables and age has shown no group differences.

**Table 5:** Demographic Questionnaire ratings of the study groups (n=40).

Variables			Group	
			Compulsive buyers	Non-compulsive buyers
Age Mean (SD)			23.95 (2.58)	24.05 (1.95)
Gender	Female	n	10	10
		%	50%	50%
	Male	n	10	10
		%	50%	50%
Religious	No	n	11	10
		%	55%	50%
	Yes	n	9	10
		%	45%	50%
Family status	Single	n	17	15
		%	85%	75%
	Married	n	3	5
		%	15%	25%
Academic education	No	n	0	3
		%	0%	15%
	Yes	n	20	17
		%	100%	85%

Compulsive Buying Scale (CBS) (34) see earlier description. In our study, the questionnaire had a Cronbach’s internal reliability of α=0.89.

Barratt Impulsiveness Scale (BIS-11) see earlier description. In our study, the questionnaire had a Cronbach’s internal reliability of α=0.77.

Sensation Seeking Scale was developed by Zuckerman, Kolin, Price & Zoob (54). The questionnaire has 4 subscales, including Thrill and Adventure Seeking, Disinhibition, Experience Seeking, and Boredom Susceptibility. Each subscale contains 10 items, making a total of 40 items. The questionnaire had a Cronbach’s internal reliability of α=0.83-0.86 (55). In our study, the questionnaire had a Cronbach’s internal reliability of α=0.75.

### 4.3 Procedure

The participants were recruited by responding to an advertisement on the university website and WhatsApp groups of students. The participants filled in the questionnaires and then performed the assignment using the Zoom software. Instructions were given to each subject individually. During the task, the subjects made a simulated purchase online. Subjects were told to log into the Asus website and screen share with the experimenters. This was done to enable the experimenters to assess the buying process. Each subject was told that they had up to 800 NIS to use on the ASOS website to make online purchases. They were told that they could make their purchase with less than the limit and did not have to use all of the money they were given. They were also instructed that there was a time limit of up to 20 minutes or until a decision was made on the final shopping cart. Each subject could choose to put items in the shopping cart. They had decided to buy the item or put it in the Wish List, which meant that they were still debating about the item and had not yet made a decision about it.

### 4.4 Ethical approval

The study was approved by the University’s Institutional Review Board (IRB, Helsinki committee). All participants signed an informed consent form.

### 4.5 Statistical and data analysis

Results were analyzed using Excel (Version 16.90; Microsoft 2024) and SPSS (IBM Corp., Armonk, NY, USA). Pearson’s chi-squared test was used to compare demographic factors such as sex, age, education, religion, and marital status. Group comparisons were done using t-tests and one-way ANOVA for the CBS, BIS-11, and SSS questionnaires.

## 5 Results

Impulsivity positively correlated with compulsive buying (*r*= 0.43; p<0.01). As predicted, sensation seeking was positively correlated with compulsive buying (*r* = 0.32; p<0.05). As expected, sensation seeking was also positively correlated with impulsivity (*r* = 0.43; p<0.01). There were no sex differences between women and men in compulsive online shopping (*t*= 0.19; p>0.05).

To test the research hypothesis that there is a difference between compulsive buyers to non-compulsive buyers in their buying behavior, we compared the time it takes for the first item to enter the shopping cart. Following the research hypotheses, it was found that the time it took to enter the first item in the shopping cart among compulsive buyers (M=4.02; SD=3.8) was faster than non-compulsive buyers (M=10.02; SD=7.7) (t=3.09; p<0.01). Secondly, we tested the hypothesis that compulsive buyers would differ from non-compulsive buyers in the final sum. Results showed that the final sum of shopping among compulsive buyers (M=736.25, SD=170.7) was higher than non-compulsive buyers (M=628.08, SD=141.5). (t =-2.18, p<0.05). **Table 6** shows a comparison in all variables between compulsive buyers and non-compulsive buyers.

**Table 6:** Characteristics of the buying experiment- a comparison of compulsive and non-compulsive buyers (n=40).

Variables	Group		*t-test*
	Compulsive Buyers	Non- compulsive Buyers	
	Mean (S.D)		
Time for the first item on a wish list (minutes)	1.03 (2.09)	2.06 (1.61)	1.15
Number of items in the wish list	3.8 (4.71)	5.35 (3.84)	1.13
Time for the first item in the shopping bag (minutes)	4.02 (3.88)	10.02 (7.77)	**3.09
Number of items in the shopping bag	5.10 (1.71)	5.25 (2.44)	0.22
Average sum of items	162.25 (52)	147.82 (61)	-0.8
Total sum of items	736 (170)	628.08 (141)	* -2.18
Time of the experiment (minutes)	46.2 (14.97)	41.94 (4.28)	-0.99

*p<0.05 **p<0.01.

A multiple regression examined the contribution of factors to the variance of compulsive buying. Compulsive buying was entered into the regression as a dependent variable, while sensation seeking and impulsivity were entered into the regression as independent variables. The results showed a significant regression [F (2, 37) = 4.77, p<.01] with a multiple correlation of 0.45 and explained variance of 20%. The values of the standardized regression coefficients (β) indicate that the contribution of impulsivity, but not sensation seeking, was significant.

## 6 Discussion

### 6.1 Major findings

The present study examined impulsivity and compulsivity among compulsive and non-compulsive buyers. In particular, the study sought to test whether compulsive buying fits into the model of behavioral addictions, which are defined based on impulsive characteristics, or into the category of obsessive-compulsive spectrum disorders, which are characterized by compulsive behaviors. Behavioral addictions often lie on a spectrum between impulsivity and compulsivity, which are positively correlated (56). The findings provided partial support for impulsivity and compulsivity in compulsive buyers. First, scores on the Barratt Impulsiveness Scale questionnaire showed higher levels of impulsivity in compulsive buyers compared to non-compulsive buyers. Additionally, scores on the Yale Brown Obsessive Compulsive Scale indicated that compulsive buyers presented high levels of compulsivity compared with non-compulsive buyers. Furthermore, compulsive buying rating correlated positively with ratings of impulsivity and compulsivity, which supports the hypothesis that compulsive buying is within an impulsive-compulsive spectrum. However, the study shows some negative results. The analysis of the Delay-Discounting Task did not show a preference for immediate versus delayed rewards in compulsive buyers, contrary to the study’s hypothesis. Additionally, on the Go/No-Go task, there was no overall higher number of commission errors, indicating an impaired response inhibition in compulsive buyers. Finally, on the Dot-Probe task, there was no evidence for selective attention to shopping-related stimuli in compulsive byers, contrary to our hypothesis.

### 6.2 Evidence for impulsivity

Our study indicates some evidence for impulsivity in performance of compulsive buyers on the Go/No-Go task and on the Dot-Probe task. The negative correlations between compulsive buying scores and average reaction times on the in the No-Go commission condition on the Go/No-Go task and on the Dot-Probe task with buying stimuli indicates that participants responded fast and impulsively. It is evidence for an impairment in response inhibition and selective attention to shopping-related stimuli which indicates impulsivity. These findings are consistent with previous studies, which showed that faster responses may reflect increased impulsivity or difficulty inhibiting a response, that are characteristics of impulsivity (57). These tasks (Go/No-Go and Dot-Probe) test the ability to avoid impulsive responses, and the results indicate that compulsive buyers displayed faster (and sometimes incorrect) responses. This means difficulty in maintaining self-control. In the context of compulsive buying, the link to impulsivity can be seen as a leading factor in immediate and reckless purchases. The findings emphasize that compulsive buying not only involves an urge to act immediately but also reflects a real difficulty in inhibiting responses, which may be a key indicator of impulsivity. Previous studies that used the dot-probe paradigm with shopping-related compared to neutral pictures did not reveal conclusive results. Vogel et al. (21) showed no differences in performance between patients with CBSD and healthy individuals. Jiang et al. (23) and Trotzke et al. (22) also did not find correlations between CBSD symptoms and performance on the delay discounting task with shopping-pictures. However, Trotzke et al. (22) reported positive correlations between the attentional bias score and CBSD symptom severity after controlling for sequence effects (order of task administration).

### 6.3 Response inhibition and attention

Furthermore, compulsive buyers presented higher commission error rates in the second part of the task the Go/No-Go task compared to non-compulsive buyers. This result indicates that when the task required sustained attention over time, the experimental group had more difficulty inhibiting their responses, which was reflected in more errors on the No-Go commission condition. It is plausible that sustained attention, which requires prolonged cognitive effort, affects the ability to inhibit and causes higher impulsivity. This interpretation is consistent with previous studies that have shown a link between sustained attention and impulsivity, particularly on tasks that require self-control over time (58). In this context, it can be assumed that the compulsive buyers’ difficulty in maintaining attention over time may stem from a combination of cognitive load and the inability to inhibit responses, which leads to the occurrence of many errors. This situation intensifies the impulsive tendency precisely at the stage when participants are faced with a prolonged demand for self-control. The finding of a lack of between-group differences in the first part of the Go/No-Go task is similar to evidence that shows that individuals with IGD display no higher error rates or perform the task less well compared to the control group (59). This finding implies that the context of the task and the duration required for it may be critical in distinguishing impulsive behavior between groups. There is therefore a need to incorporate relevant stimuli, such as shopping images versus control images, or to add social cues, to highlight the difficulties in response inhibition among populations with compulsive buying behaviors (16).

Previous experimental studies that have used response inhibition tasks in CBSD reported mixed findings. Poor response inhibition was associated with symptoms of CBSD in two studies that used the Stroop Task or the Go/No-Go Task (17, 18), while others reported no correlations between task performance and symptoms of CBSD using the Stop Signal Task (60). Hague et al. (61) reported that individuals with high symptom severity of CBSD performed worse on the Go/No-Go task using shopping-related versus neutral cues. There were no differences in task performance between patients with CBSD and control participants (21). Finally, a modified affective shifting task showed interactions between implicit cognitive processes (attentional bias and implicit associations), craving responses, and inhibitory control performance on symptom severity of CBSD (22). These mixed findings have led Thomas et al. (16) to conclude that there is a lack of evidence or conflicting evidence for impairment of delay discounting, Stroop, trail-making, Iowa Gambling Task (IGT), Go/No-Go, and stop-signal tasks in individuals with BSD.

### 6.4 Delay discounting

In the Delay-Discounting task, no differences in delay discounting (K) were found between groups. This discrepancy is consistent with the methodological problem presented in previous studies, which found that the relationship between Delay-Discounting and impulsive behavior is not always consistent, especially when it comes to clinical groups such as behavioral addictions. Possible reasons for this discrepancy include the structure of the Delay-Discounting task, which does not distinguish between different types of impulsive behaviors (e.g., compulsive buying versus gambling or drug use (62). Nikolai and Moshagen (19) examined impulsive behaviors in compulsive buying using a Delay-Discounting task. In their study, the researchers offered immediate rewards in amounts ranging from $10 to $100, spread over different periods. They found that CB predicted steeper discounting functions on the DDT over and above the remaining symptom measures and trait impulsivity. The differences in findings may be due to the small amounts of money offered in the current task (1.20 shekels delayed and uncertain, versus a small, immediate sum of money). Frye and colleagues (63) noted that matching the amount of money to the research topic is critical to ensuring a true reflection of the subjects’ behaviors. Small amounts relative to the amounts involved in real-life buying cause subjects not to perceive the differences between the postponement options optimally, or not to evaluate the future reward as sufficiently interesting. This result may reduce the chance of detecting a significant between-group difference. When the amounts offered do not match the financial amounts that subjects are used to when shopping, this may significantly reduce their motivation to perform the task, thus reducing the chance of identifying a significant difference (63).

### 6.5 Anxiety and depression

The current study found a significant positive association between compulsive buying behavior and anxiety and depression levels, as measured by the STAI and BDI. These findings align with previous research indicating comorbidity between compulsive buying and psychiatric disorders (3–5). However, no significant group differences were found in trait anxiety and depression levels. While group differences in state anxiety were only marginally significant, the medium effect size (Cohen’s d = 0.32) suggests a potentially meaningful relationship. This partial significance may be due to the relatively small sample size or to the exclusion of participants with psychiatric diagnoses, which may have reduced variability in distress-related measures.

### 6.6 Simulation of buying/shopping online

Our following experiment has simulated a natural buying experiment online, which tested impulsivity and delay of gratification. The study findings showed a positive relationship between impulsivity, sensation-seeking and compulsive buying. Furthermore, individuals with compulsive buying showed an impairment in delayed gratification indicated by faster times taken to add items to the shopping cart and that they spent more money than the control group. Finally, the regression analysis has shown that impulsivity but not sensation seeking, contributed to the variance of compulsive buying. The results she some light on the process of impulsive buying. The difference between a planned and unplanned purchase is that the product is searched for and details about it are found out in advance, compared to an impulsive purchase that occurs in a quick decision-making process. Researchers have classified impulsive purchases as an unplanned purchase, characterized by making a purchase quickly, followed by emotional and cognitive reactions, and where the behaviors that cause these reactions are themselves also emotional. According to Zhang, Cheng and Huang (64), impulse purchasing occurs when a buyer acts upon an impulse and purchases an item without a previous plan. This behavior is characterized by its spontaneous nature. According to Zhang and Ahmad (65), impulse buying may be characterized by four characteristics: unplanned purchase, it is triggered by a particular stimulus, like an advertisement or a promotion, the decision is spontaneous and the act of purchasing is driven by both cognitive and emotional factors.

### 6.7 Sensation seeking and compulsive buying

The current study found that although sensation-seeking correlated with impulsivity and compulsive buying, it did not contribute to ratings on compulsive buying according to the regression model. The evidence for the relationship between sensation seeking and CB is mixed. Williams and Grisham (66) found no correlations between sensation seeking and CB. However, when sensation seeking was assessed as a separate entity from impulsivity using the Brief Sensation Seeking Scale, there was a significant difference between compulsive buyers and non-compulsive buyers (53). Similarly, sensation seeking scores followed an upward trend across levels of compulsive buying groups (below average, moderately, and above average on CB (52). It seems that when sensation seeking is captured as a unique construct, separate from impulsivity, it is more likely to be positively associated with CB, however, it was not the case in our study.

### 6.8 Limitations and future directions

This is a cross-sectional study, so no inferences about causality can be made. Secondly, there was little correlation between subjective ratings of impulsivity or compulsivity and cognitive tasks’ performance, suggesting that these examine different cognitive mechanisms. Self-report questionnaires and behavioral tasks assess distinct facets of impulsivity. Questionnaires tend to reflect cognitive and emotional components, while behavioral tasks measure response inhibition and control. Therefore, discrepancies between these measures are expected and highlight the multifaceted nature of impulsivity rather than inconsistency. Other factors that may have influenced the results may be demand characteristics. Participants may have chosen to answer the questionnaire in a way that would present themselves in a positive light, or to be accepted into the study and receive rewards, which could influence the study results and create unintended biases. Furthermore, the sample was based on a student population (psychology undergraduates), which may limit the generalizability of the findings to broader populations. The sample also exhibited a gender imbalance (81% female) and predominantly low-income backgrounds, which may further constrain external validity. It is recommended in the future to increase the sample size and expand the study to different populations, such as different ages, populations with different cultural characteristics, or people with different intensities of compulsive buying behavior. Additionally, future studies could utilize additional tasks tailored to the specific context of compulsive buying, such as tasks that simulate real-world shopping experiences. These tasks may improve the relevance of the measurements and be closer to real-world compulsive behaviors, which could add empirical value to the field.

## 7 Conclusions

The study confirms previous evidence for associations between compulsive buying, impulsivity, and compulsivity. The high levels of impulsivity and compulsiveness found in the experimental group support the classification of the behavior under both behavioral addictions and impulse control disorders. Although several fundamental hypotheses, such as those concerning delay discounting and selective attention, were not supported by the data, our study made several new contributions. First, it uniquely showed impaired inhibition with a higher cognitive load (but not with low cognitive load) in compulsive buyers, indicated by their performance on the Go/No-Go task. This evidence implies that compulsive buyers’ difficulty in maintaining attention over time may stem from a combination of cognitive load and the inability to inhibit responses Secondly, negative correlations between compulsive buying scores and average reaction times on the in the No-Go commission condition on the Go/No-Go task and on the Dot-Probe task with buying stimuli indicate that participants responded fast and impulsively. Third, although there was no evidence for impaired delay discounting of low rewards in compulsive buyers, in a simulation of real-life shopping, compulsive buyers were faster to choose items, and they paid more for them. This evidence suggests that the effects of compulsive buying on cognitive function are often subtle; the way forward is to use a real-life simulation that uniquely demonstrates this impairment.

## Data Availability

The raw data supporting the conclusions of this article will be made available by the authors, without undue reservation.

## References

[1] Weinstein A Maraz A Griffiths MD Lejoyeux M Demetrovics Z. Compulsive buying features and characteristics of addiction. In: Neuropathology of drug addictions and substance misuse. USA: Academic Press (2016). p. 993–1007.

[2] Müller A Mitchell JE de Zwaan M. Compulsive buying. Am J Addict. (2015) 24:132–7. doi: 10.1111/ajad.12111, PMID: 25864601

[3] Lejoyeux M Tassain V Solomon J Ades J. Study of compulsive buying in depressed patients. J Clin Psychiatry. (1997) 58:169–73. doi: 10.4088/JCP.v58n0406, PMID: 9164428

[4] Müller A Mitchell JE Black DW Crosby RD Berg K de Zwaan M. Latent profile analysis and comorbidity in a sample of individuals with compulsive buying disorder. Psychiatry Res. (2010) 178:348–53. doi: 10.1016/j.psychres.2010.04.021, PMID: 20471099

[5] Lejoyeux M Bailly F Moula H Loi S Adès J. Study of compulsive buying in patients presenting obsessive-compulsive disorder. Compr Psychiatry. (2005) 46:105–10. doi: 10.1016/j.comppsych.2004.07.027, PMID: 15723026

[6] Black DW Repertinger S Gaffney GR Gabel J. Family history and psychiatric comorbidity in persons with compulsive buying: preliminary findings. Am J Psychiatry. (1998) 155:960–3. doi: 10.1176/ajp.155.7.960, PMID: 9659864

[7] Weinstein A Mezig H Mizrachi S Lejoyeux M. A study investigating the association between compulsive buying with measures of anxiety and obsessive–compulsive behavior among internet shoppers. Compr Psychiatry. (2015) 57:46–50. doi: 10.1016/j.comppsych.2014.11.003, PMID: 25465653

[8] Maraz A Griffiths MD Demetrovics Z. The prevalence of compulsive buying: a meta-analysis. Addiction. (2016) 111:408–19. doi: 10.1111/add.13223, PMID: 26517309

[9] Black D W. Compulsive shopping as a behavioral addiction. In: Petry N (Ed). Behavioral Addictions: DSM-5® and Beyond. Oxford University Press, Oxford; New York (2015) pp 125–156.

[10] American Psychiatric Association. Diagnostic and statistical manual of mental disorders, 5th. (Washington D.C USA: American Psychiatric Association) (2022). doi: 10.1176/appi.books.9780890425787., PMID:

[11] World Health Organization. International statistical classification of diseases and related health problems (2019). Available online at: https://icd.who.int/ (Accessed July 25, 2025).

[12] Trotzke P Brand M Starcke K. Cue-reactivity, craving, and decision making in buying disorder: A review of the current knowledge and future directions. Curr Addict Rep. (2017) 4:246–53. doi: 10.1007/s40429-017-0155-x

[13] Müller A Brand M Claes L Demetrovics Z de Zwaan M Fernández-Aranda F. Buying-shopping disorder-is there enough evidence to support its inclusion in ICD-11? CNS Spectrums. (2019) 24:374–9. doi: 10.1017/S1092852918001323, PMID: 30604662

[14] Müller A Laskowski NM Trotzke P Ali K Fassnacht DB De Zwaan M. Proposed diagnostic criteria for compulsive buying-shopping disorder: A Delphi expert consensus study. J Behav Addict. (2021) 10:208–22. doi: 10.1556/2006.2021.00013, PMID: 33852420 PMC8996806

[15] McElroy SL Keck PE Pope HG Smith JM Strakowski SM. Compulsive buying: A report of 20 cases. J Clin Psychiatry. (1994) 55:242–8., PMID: 8071278

[16] Thomas TA Joshi M Trotzke P Steins-Loeber S Müller A. Cognitive functions in compulsive buying-shopping disorder: A systematic review. Curr Behav Neurosci Rep. (2023) 10:1–19. doi: 10.1007/s40473-023-00255-6

[17] Nicolai J Daranc S Moshagen M. Effects of mood state on impulsivity in pathological buying. Psychiatry Res. (2016) 244:351–6. doi: 10.1016/j.psychres.2016.08.009, PMID: 27521976

[18] Lindheimer N Nicolai J Moshagen M. General rather than specific: cognitive deficits in suppressing task irrelevant stimuli are associated with buying-shopping disorder. PloS One. (2020) 15:e0237093. doi: 10.1371/journal.pone.0237093, PMID: 32750087 PMC7402500

[19] Nicolai J Moshagen M. Dissociating pathological buying from obsessive-compulsive symptoms using delay discounting. Z Für Psychol. (2017) 225:244–51. doi: 10.1027/2151-2604/a000308

[20] Derbyshire KL Chamberlain SR Odlaug BL Schreiber L Grant JE. Neurocognitive functioning in compulsive buying disorder. Ann Clin Psychiatry. (2014) 26:57–63. doi: 10.5194/gi-2016-11-RC2, PMID: 24501731

[21] Vogel B Trotzke P Steins-Loeber S Schefer G Stenger J de Zwaan M. An experimental examination of cognitive processes and response inhibition in patients seeking treatment for buying-shopping disorder. PloS One. (2019) 14:e0212415. doi: 10.1371/journal.pone.0212415, PMID: 30840643 PMC6402626

[22] Trotzke P Müller A Brand M Starcke K Steins-Loeber S. Buying despite negative consequences: interaction of craving, implicit cognitive processes, and inhibitory control in the context of buying-shopping disorder. Addictive Behav. (2020) 110:106523. doi: 10.1016/j.addbeh.2020.106523, PMID: 32652388

[23] Jiang Z Zhao X Li C. Self-control predicts attentional bias assessed by online shopping-related Stroop in high online shopping addiction tendency college students. Compr Psychiatry. (2017) 75:14–21. doi: 10.1016/j.comppsych.2017.02.007, PMID: 28284828

[24] Field M Cox WM. Attentional bias in addictive behaviors: a review of its development, causes, and consequences. Drug Alcohol Depend. (2008) 97:1–20. doi: 10.1016/j.drugalcdep.2008.03.030, PMID: 18479844

[25] Ryan F. Detected, selected, and sometimes neglected: cognitive processing of cues in addiction. Exp Clin Psychopharmacol. (2002) 10:67. doi: 10.1037/1064-1297.10.2.67, PMID: 12022800

[26] Fineberg NA Chamberlain SR Goudriaan AE Stein DJ Vanderschuren LJ Gillan CM. New developments in human neurocognition: clinical, genetic, and brain imaging correlates of impulsivity and compulsivity. CNS Spectr. (2014) 19:69–89. doi: 10.1017/S1092852913000801, PMID: 24512640 PMC4113335

[27] Black DW Shaw M McCormick B Bayless JD Allen J. Neuropsychological performance, impulsivity, ADHD symptoms, and novelty seeking in compulsive buying disorder. Psychiatry Res. (2012) 30:200(2–3):581-7. doi: 10.1016/j.psychres.2012.06.003, PMID: 22766012 PMC3665329

[28] Trotzke P Starcke K Pedersen A Müller A Brand M. Impaired decision making under ambiguity but not under risk in individuals with pathological buying-behavioral and psychophysiological evidence. Psychiatry Res. (2015) 229:551–8. doi: 10.1016/j.psychres.2015.05.043, PMID: 26165961

[29] Rachlin H Raineri A Cross D. Subjective probability and delay. J Exp Anal Behav. (1991) 55:233–44. doi: 10.1901/jeab.1991.55-233, PMID: 2037827 PMC1323057

[30] Gomez P Ratcliff R Perea M. A model of the go/no-go task. J Exp Psychol Gen. (2007) 136:389–413. doi: 10.1037/0096-3445.136.3.389, PMID: 17696690 PMC2701630

[31] Goodman WK Price LH Rasmussen SA Mazure C Fleiscmann RL Hill CL. The Yale-Brown Obsessive Compulsive Scale (YBOCS): Part I. Development, use, and reliability. Arch Gen Psychiatry. (1989) 46:1006–11. doi: 10.1001/archpsyc.1989.01810110048007, PMID: 2684084

[32] Beck AT Ward C Mendelson M Mock J Erbaugh J. J. A. G. P. Beck depression inventory (BDI). Arch Gen Psychiatry. (1961) 4:561–71. doi: 10.1001/archpsyc.1961.01710120031004, PMID: 13688369

[33] Spielberger CD Gorsuch RL Lushene R Vagg PR Jacobs GA. Manual for the state-trait anxiety inventory. Manual for the state-trait anxiety inventory. Palo Alto, CA: Consulting Psychologists Press (1983).

[34] Valence G d’Astous A Fortier L. Compulsive buying: Concept and measurement. J Consumer Policy. (1988) 11:419–33. doi: 10.1007/BF00411854

[35] Kyriazos TA. Applied psychometrics: Sample size and sample power considerations in factor analysis (EFA, CFA) and SEM in general. Psychology. (2018) 9:2207. doi: 10.4236/psych.2018.98126

[36] Faber RJ O’guinn TC. A clinical screener for compulsive buying. J Consumer Res. (1992) 19:459–69. doi: 10.1086/209315

[37] Reisch L Gwozdz W Raab G. Compulsive buying in Denmark: The first study on Danish consumers’ tendency to compulsive buying. J Consumer Policy. (2011) 11:419–33.

[38] Kaur M Maheshwari SK Kumar A. Compulsive buying behavior and online shopping addiction among health science teachers. Int J Nurs Care. (2019) 7:74–80. doi: 10.5958/2320-8651.2019.00014.0

[39] Patton JH Stanford MS Barratt ES. Factor structure of the Barratt impulsiveness scale. J Clin Psychol. (1995) 51:768–74. doi: 10.1002/1097-4679(199511)51:6<768::AID-JCLP2270510607>3.0.CO;2-1 8778124

[40] Beck A T Steer R A Carbin M G. Psychometric properties of the Beck Depression Inventory: Twenty-five years of evaluation. Clin Psychol Rev. (1988) 8(1):77–100. doi: 10.1016/0272-7358(88)90050-5

[41] Saville BK Gisbert A Kopp J Telesco C. Internet addiction and delay discounting in college students. The. psychol Records. (2010) 60:273–86. doi: 10.1007/BF03395707

[42] Reynolds B Schiffbauer R. Measuring state changes in human delay discounting: An experiential discounting task. Behav Processes. (2004) 67:343–56. doi: 10.1016/j.beproc.2004.06.003, PMID: 15518985

[43] Mazur JE. An adjusting procedure for studying delayed reinforcement. In: Commons ML Mazur JE Nevin JA Rachlin H, editors. Quantitative Analysis of Behavior: Vol 5. The Effects of Delay and Intervening Events on Reinforcement Value. Erlbaum, Hillsdale, NJ: Lawrence Elbaum Associates, INC (1987). p. 55–73.

[44] Rachlin H. The science of self-control. Cambridge, MA: Harvard University Press (2000).

[45] Richards JB Zhang L Mitchell SH De Wit H. Delay or probability discounting in a model of impulsive behavior: Effect of alcohol. J Exp Anal Behav. (1999) 71:121–43. doi: 10.1901/jeab.1999.71-121, PMID: 10220927 PMC1284697

[46] MacLeod C Mathews A Tata P. Attentional bias in emotional disorders. J Abnormal Psychol. (1986) 95:15. doi: 10.1037/0021-843X.95.1.15 3700842

[47] JASP Team. JASP (Version 0.18.3) [Computer software] (2024). Available online at: https://jasp-stats.org (Accessed June 10, 2025).

[48] Faul F Erdfelder E Buchner A Lang A-G. Statistical power analyses using G^*^Power 3.1: Tests for correlation and regression analyses. Behav Res Methods. (2009) 41:1149–60. doi: 10.3758/BRM.41.4.1149, PMID: 19897823

[49] Kang H. Sample size determination and power analysis using the G*Power software. J Educ Eval Health Professions. (2021) 18:17. doi: 10.3352/jeehp.2021.18.17, PMID: 34325496 PMC8441096

[50] Meshi D Elizarova A Bender A Verdejo-Garcia A. Excessive social media users demonstrate impaired decision making in the Iowa Gambling Task. J Behav Addict. (2019) 8(1):169–173. doi: 10.1556/2006.7.2018.138, PMID: 30626194 PMC7044593

[51] Zuckerman M. Behavioral expressions and biosocial bases of sensation seeking. Cambridge University Press (1994).

[52] Rodríguez-Villarino R González-Lorenzo M Fernández-González Á. Lameiras-Fernández M Foltz ML. Individual factors associated with buying addiction: an empirical study. Addict Res Theory. (2006) 14:511–25. doi: 10.1080/16066350500527979

[53] Maraz A van den Brink W Demetrovics Z. Prevalence and construct validity of compulsive buying disorder in shopping mall visitors. Psychiatry Res. (2015) 228:918–924. doi: 10.1016/j.psychres.2015.04.012, PMID: 26027442

[54] Zuckerman M Kolin EA Price L Zoob I. Development of a sensation-seeking scale. J Consult Psychol. (1964) 28:477–82. doi: 10.1037/h0040995, PMID: 14242306

[55] Zuckerman M Aluja A. Measures of sensation seeking. In: Boyle GJ Saklofske DH Matthews G, editors. Measures of Personality and Social Psychological Constructs. Academic Press, Oxford (2014). p. 352–80.

[56] Grant JE Potenza MN Weinstein A Gorelick DA. Introduction to behavioral addictions. Am J Drug Alcohol Abuse. (2010) 36:233–41. doi: 10.3109/00952990.2010.491884, PMID: 20560821 PMC3164585

[57] Proust J. Time and action: Impulsivity, habit, strategy. Rev Philosophy Psychol. (2015) 6:717–43. doi: 10.1007/s13164-014-0224-1

[58] O’Connell RG Dockree PM Bellgrove MA Turin A Ward S Foxe JJ. Two types of action error: Electrophysiological evidence for separable inhibitory and sustained attention neural mechanisms producing error on go/no-go tasks. J Cogn Neurosci. (2009) 21:93–104. doi: 10.1162/jocn.2009.21008, PMID: 18476764

[59] Lee RS Hoppenbrouwers S Franken I. A systematic meta-review of impulsivity and compulsivity in addictive behaviors. Neuropsychol Rev. (2019) 29:14–26. doi: 10.1007/s11065-019-09402-x, PMID: 30927147

[60] Billieux J Gay P Rochat L van der Linden M. The role of urgency and its underlying psychological mechanisms in problematic behaviours. Behav Res Ther. (2010) 48:1085–96. doi: 10.1016/j.brat.2010.07.008, PMID: 20708729

[61] Hague B Kellett S Sheeran P. Testing the generalizability of impulse control problems in compulsive buying. J Soc Clin Psychol. (2016) 35:269–88. doi: 10.1521/jscp.2016.35.4.269

[62] Moreira D Barbosa F. Delay discounting in impulsive behavior: A systematic review. Eur Psychol. (2019) 24:312–21. doi: 10.1027/1016-9040/a000360

[63] Frye CC Galizio A Friedel JE DeHart WB Odum AL. Measuring delay discounting in humans using an adjusting amount task. J Visualized Experiments. (2016) 107):53584. doi: 10.3791/53584, PMID: 26779747 PMC4781322

[64] Zhang X Cheng X Huang X. Oh, My God, Buy It!” Investigating impulse buying behavior in live streaming commerce. Int J Human–Computer Interaction. (2023) 39:2436–49. doi: 10.1080/10447318.2022.2076773

[65] Zhang Q Ahmad W. Online impulse purchase in social commerce: roles of social capital and information overload. Int J Human–Computer Interaction. (2023) 40(2):1–18. doi: 10.1080/10447318.2023.2212862

[66] Williams AD Grisham JR. Impulsivity, emotion regulation, and mindful attentional focus in compulsive buying. Cogn Ther Res. (2011) 36:451–7. doi: 10.1007/s10608-011-9384-9

